# A *Drosophila* Pattern Recognition Receptor Contains a Peptidoglycan Docking Groove and Unusual L,D-Carboxypeptidase Activity

**DOI:** 10.1371/journal.pbio.0020277

**Published:** 2004-09-07

**Authors:** Chung-I Chang, Sébastien Pili-Floury, Mireille Hervé, Claudine Parquet, Yogarany Chelliah, Bruno Lemaitre, Dominique Mengin-Lecreulx, Johann Deisenhofer

**Affiliations:** **1**Howard Hughes Medical Institute and Department of Biochemistry, University of Texas Southwestern Medical CenterDallas, Texas, United States of America; **2**Centre de Génétique Moléculaire du Centre National de la Recherche ScientifiqueGif-sur-Yvette, France; **3**Institut de Biochimie et Biophysique Moléculaire et Cellulaire, Centre National de la Recherche ScientifiqueUniversité de Paris-Sud, OrsayFrance

## Abstract

The *Drosophila* peptidoglycan recognition protein SA (PGRP-SA) is critically involved in sensing bacterial infection and activating the Toll signaling pathway, which induces the expression of specific antimicrobial peptide genes. We have determined the crystal structure of PGRP-SA to 2.2-Å resolution and analyzed its peptidoglycan (PG) recognition and signaling activities. We found an extended surface groove in the structure of PGRP-SA, lined with residues that are highly diverse among different PGRPs. Mutational analysis identified it as a PG docking groove required for Toll signaling and showed that residue Ser158 is essential for both PG binding and Toll activation. Contrary to the general belief that PGRP-SA has lost enzyme function and serves primarily for PG sensing, we found that it possesses an intrinsic L,D-carboxypeptidase activity for diaminopimelic acid-type tetrapeptide PG fragments but not lysine-type PG fragments, and that Ser158 and His42 may participate in the hydrolytic activity. As L,D-configured peptide bonds exist only in prokaryotes, this work reveals a rare enzymatic activity in a eukaryotic protein known for sensing bacteria and provides a possible explanation of how PGRP-SA mediates Toll activation specifically in response to lysine-type PG.

## Abstract 2

The Drosophila protein PGRP-SA is found to have an intrinsic activity to cleave L,D-configured peptide bonds, which exist only in prokaryotes. This work reveals a rare enzymatic activity in a eukaryotic protein known for sensing bacteria

## Introduction

Activation of innate immunity in response to bacterial pathogens requires a group of molecules, known as the pattern recognition receptors, that recognize conserved motifs, present in bacteria but absent in higher eukaryotes, and trigger downstream signaling events. In *Drosophila,* two distinct signal transduction pathways are involved in the pathogen-specific innate immune response by inducing the expression of a panel of specific antimicrobial peptides (Tzou et al. 2002; Hoffmann 2003). The Toll signaling pathway responds mainly to Gram-positive bacterial or fungal infections, which lead to the proteolytic processing of the cytokine-like polypeptide Spätzle. Binding of the cleaved Spätzle to the transmembrane receptor Toll activates an intracellular signaling cascade that results in the degradation of the IκB-like protein Cactus and the nuclear localization of the NF-κB–like proteins Dif and Dorsal, which induce the transcription of several antimicrobial peptide genes, such as *Drosomycin* (Lemaitre et al. 1996, 1997; Meng et al. 1999; Rutschmann et al. 2000b; Tauszig-Delamasure et al. 2002; Weber et al. 2003). By contrast, the immune deficiency (Imd) pathway mediates defense reactions against primarily Gram-negative bacteria through different signaling components and regulates the cleavage and activation of another NF-κB–related nuclear factor, Relish, which activates a different set of antimicrobial peptide genes, including *Diptericin* (Lemaitre et al. 1995; Hedengren et al. 1999; Leulier et al. 2000; Rutschmann et al. 2000a; Vidal et al. 2001).

Several genetics studies have shown that the Toll pathway and the Imd pathway are activated specifically by two distinct peptidoglycan recognition proteins (PGRPs) in response to bacterial infections (Michel et al. 2001; Choe et al. 2002; Gottar et al. 2002; Ramet et al. 2002). PGRPs constitute a highly diversified family of proteins present in both insects and mammals. Members of the PGRP family are expressed as either secreted, cytosolic, or transmembrane forms, which all share a conserved 165-amino acid domain (the PGRP domain) with an evolutionary connection to bacteriophage T7 lysozyme (Yoshida et al. 1996; Kang et al. 1998; Ochiai and Ashida 1999; Werner et al. 2000; Liu et al. 2001). There are 13 PGRP genes in the genome of *Drosophila* (Werner et al. 2000). Remarkably, a gene knockout of PGRP-SA, an extracellular protein, is sufficient to eliminate Toll activation in response to the Gram-positive bacterium Micrococcus luteus in adult flies (Michel et al. 2001). Similar loss-of-function screenings have identified PGRP-LC as the surface transmembrane receptor for the Imd pathway, although another PGRP member, PGRP-LE, may also be involved in Imd activation (Choe et al. 2002; Gottar et al. 2002; Ramet et al. 2002; Takehana et al. 2002; Werner et al. 2003).

Several PGRPs have been shown to bind peptidoglycan (PG) (Yoshida et al. 1996; Werner et al. 2000; Takehana et al. 2002; Kim et al. 2003), an essential and unique cell-wall polymer found in both Gram-positive and Gram-negative bacteria. PG is composed of long glycan chains made of two alternating sugars and cross-linked by short peptides. The subunits of PG, also known as muropeptides, are composed of *N*-acetyl glucosamine (GlcNAc) and *N*-acetyl muramic acid (MurNAc) plus a stem peptide chain consisting of D- and L- (or *meso*-) amino acids, with the third amino acid being most frequently lysine in Gram-positive bacteria and diaminopimelic acid (DAP) in Gram-negative bacteria. Recently, Leulier and colleagues (2003) have shown that the Toll pathway is activated primarily by lysine-type PG found in most Gram-positive bacteria but responds weakly to DAP-type PG from Gram-negative bacteria. Not only did this finding reinforce the identification of PGRP-SA and PGRP-LCs as the putative receptors of the Toll and Imd pathways for bacterial molecular patterns, respectively, it also suggested that the signaling specificities of these two pathways might rely on the binding capability of the two activating PGRPs towards specific PG forms.

## Results/Discussion

**Figure 1:**
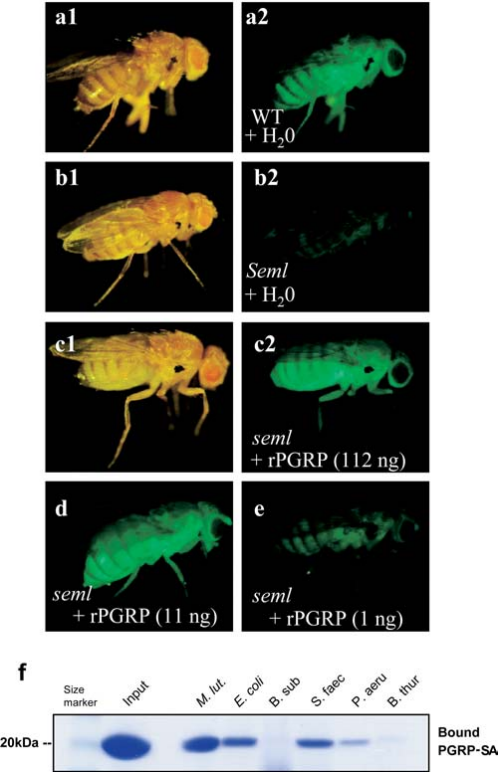
The In Vivo Rescuing and In Vitro PG-Binding Activities of Wild-Type rPGRP-SA (A–E) *Drosomycin-GFP* expression in (A) wild-type and (B to E) *PGRP-SA^seml^* flies after challenge by *M. luteus.* (A and B) Water or (C to E) rPGRP-SA at variable concentrations was injected into *Drosomycin-GFP* flies prior to the challenge with *M. luteus.* (F) rPGRP-SA binds to both lysine-type *(M. luteus* and *E. faecalis)* and DAP-type *(E. coli* and *P. aeruginosa)* PGs but not to amidated DAP-type *(Bacillus subtilis* and *Bacillus thuringiensis)* PG. The left lane (Input) is loaded with the same amount (20 μg) of protein used for the binding assay.

To facilitate molecular characterization of PG recognition and signal transduction mediated by PGRP-SA, we overexpressed and purified recombinant PGRP-SA (rPGRP-SA) in a baculovirus-insect cell expression system. PGRP-SA is a secreted protein circulating in the hemolymph (the insect blood) of *Drosophila.* We tested the activity of rPGRP-SA in vivo by injecting the protein into wild-type *(wt)* and PGRP-SA–deficient *(PGRP-SA^seml^)* flies. For this assay we used flies carrying a *Drosomycin-GFP* reporter transgene, which served as the target gene of the Toll signaling pathway. The *wt* flies injected with water produced *Drosomycin-GFP* after challenge by *M. luteus,* whereas *PGRP-SA^seml^* flies failed to express the reporter gene after the same treatment (Figure 1A and 1B). When 112 ng of rPGRP-SA was injected into *PGRP-SA^seml^* flies, the recipient flies became capable of producing *Drosomycin-GFP* after challenge with M. luteus (Figure 1C). As little as 11 ng of rPGRP-SA was sufficient to rescue *PGRP-SA^seml^* flies (Figure 1D). Injection of 11 ng of rPGRP-SA in *wt* and *PGRP-SA^seml^* flies without any further microbial challenge could not activate *Drosomycin-GFP* expression (unpublished data). These results demonstrate that rPGRP-SA expressed in insect cell culture medium is active in vivo.

The selective activation of the Toll and Imd pathways by distinct classes of bacteria is mediated via recognition of specific forms of PGs (Leulier et al. 2003). We analyzed the PG binding of rPGRP-SA to test whether the differential activation of Toll by different PG forms reflects their different binding ability towards PGRP-SA. We found that rPGRP-SA binds to purified lysine-type PGs from M. luteus or Enterococcus faecalis and to DAP-type PGs from Escherichia coli or *Pseudomonas aeruginosa,* but not to amidated DAP-type PGs from *Bacillus* (Figure 1F). Although the sensitivity of this assay is insufficient to compare the differential binding of rPGRP-SA to lysine- and DAP-type PGs, these results are overall in good agreement with previous in vivo challenge data (Leulier et al. 2003). Unlike lysine-type PGs, DAP-type PGs can only weakly activate the Toll pathway, which may be explained by the unexpected hydrolyzing activity of PGRP-SA for DAP-type PGs, as described below.

**Figure 2:**
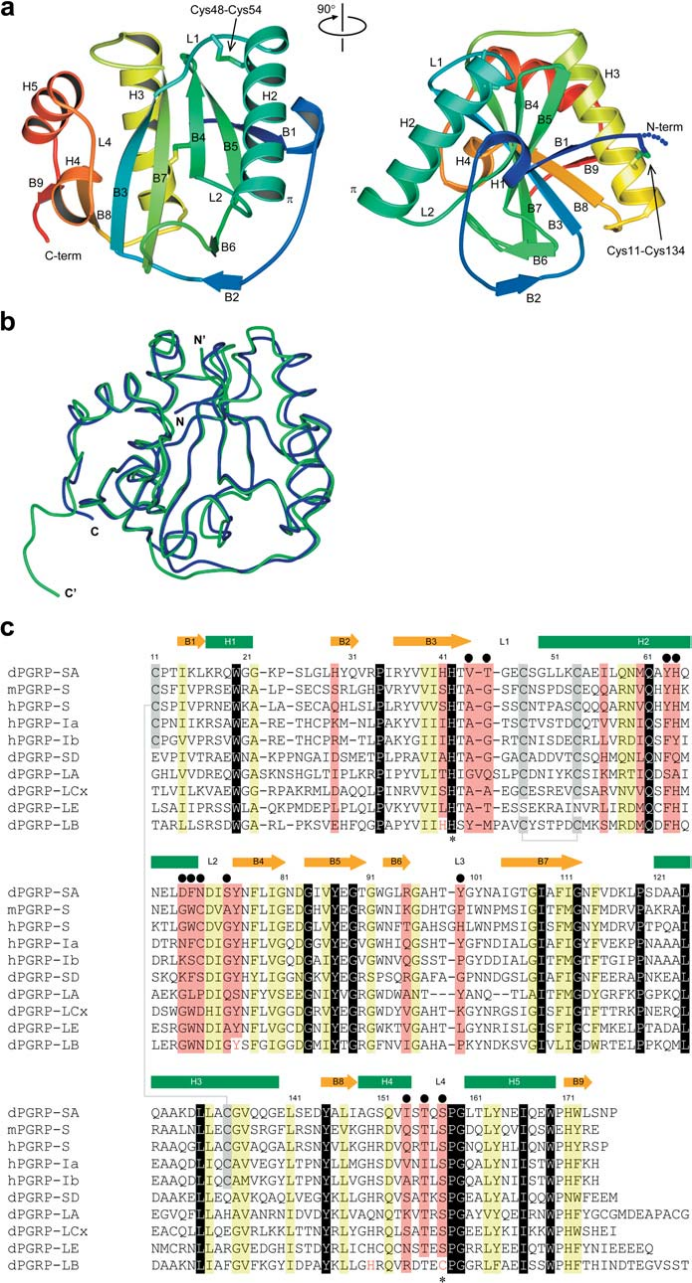
PGRP-SA Structure and Sequence Comparisons (A) Ribbon diagram showing the front view (left) and side view (right) of PGRP-SA. The ribbon is colored from N to C terminus in a progression from blue to red. Disulfide bridges are shown as sticks. The π helix turn at the end of the H2 helix is indicated. (B) Comparison of PGRP-SA (blue coil, from N to C) and PGRP-LB (green coil, from N′ to C′). (A) and (B) were prepared with Bobscript (Esnouf 1999), GL_RENDER (E. Esser, personal communication), and POV-Ray (Persistence of Vision Ray Tracer v3.1g). (C) Aligned sequences of selected PGRP domains, with a serine and a histidine at position 158 and position 42 of PGRP-SA (marked with asterisks), respectively, from *Drosophila* (d), mouse (m), and human (h). Secondary-structure elements in PGRP-SA are indicated above the alignment. Invariant residues are boxed in black and colored in white, conserved residues are shaded in yellow, and those lining the putative PG docking groove are in pink. The disulfide bond-forming Cys residues are boxed in gray. The residue number of PGRP-SA is shown above the alignment. The residues chosen for mutagenesis are marked with black circles. A structurally based alignment of the dPGRP-LB sequence is shown at the bottom with its amidase catalytic zinc-coordinating residues colored in red.

**Figure 3:**
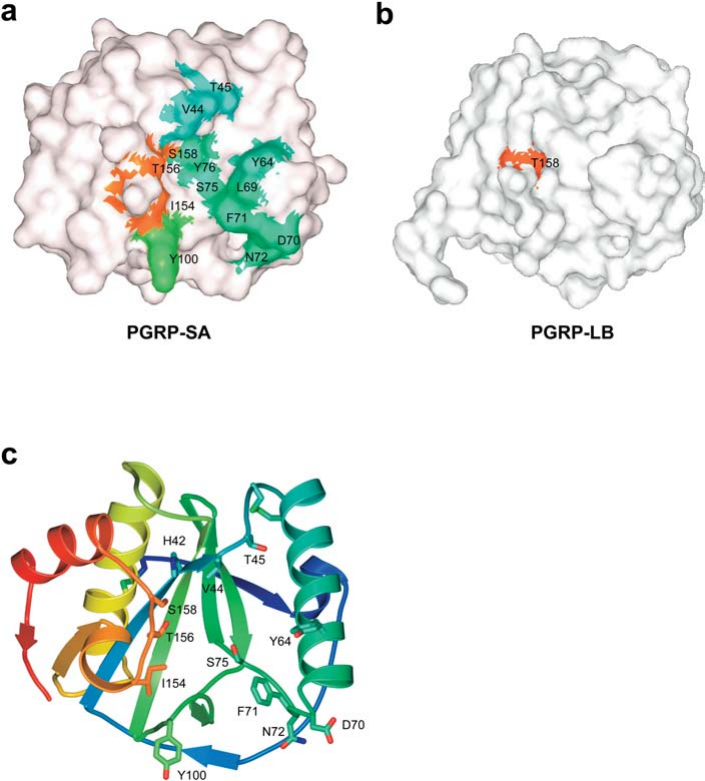
Structural Analysis of PGRP-SA (A and B) Molecular surfaces of (A) PGRP-SA and (B) PGRP-LB shown in similar orientations. Selected PGRP-SA residues on the putative PG docking groove are highlighted on the surface. Thr158 of PGRP-LB, the residue corresponding to Thr156 of PGRP-SA, is highlighted for reference. (C) Stick model of the PGRP-SA residues chosen for mutational analysis. Residues are colored with the same rainbow-coloring scheme as in Figure 2A. Figures were prepared with GRASP (Nicholls et al. 1991), Bobscript, GL_RENDER, and POV-Ray.

We crystallized PGRP-SA using seeding methods, and collected complete data to 2.2-Å resolution from a single crystal plate at the SBC 19-ID beamline of the Advanced Photon Source (APS) at Argonne National Laboratory. The crystal structure was determined by molecular replacement, using the structure of PGRP-LB as a search model. PGRP-LB is a zinc amidase similar in structure to T7 lysozyme (Kim et al. 2003) and is 29% identical in amino acid sequence to PGRP-SA. Although the crystals were grown from full-length PGRP-SA (177 residues plus 6×His-tag; residues numbered from the N terminus of the purified polypeptide chain, as determined by N-terminal sequencing), clear electron density was visible only from Cys11 to Pro177. The structure of PGRP-SA reveals a single domain composed of a central seven-stranded mixed β sheet (B1, B3, B4, B5, B7, B8, and B9) flanked by three major helices (H2, H3, and H5), a small two-stranded parallel β sheet (B2 and B6), and two single-turn helices (H1 and H4) (Figure 2A). The H2 helix contains one turn of rarely observed π helix at its C terminus (residues 64–70). This helix, together with the L1–L4 loops and the central β sheet, forms a prominent extended surface groove (Figure 2A and 3), which in PGRP-LB includes a zinc cage (Kim et al. 2003). The overall structure of PGRP-SA strongly resembles that of PGRP-LB (Figure 2B). The root-mean-square deviation (r.m.s.d.) of the 167 Cα positions after superposition is 1.22 Å. However, PGRP-SA has lost two of the four zinc-coordinating residues present in PGRP-LB (Figure 2C); accordingly, the rPGRP-SA crystals exhibit no X-ray absorption at the zinc edge, and we found no electron density for possible metal ions around the groove. Other major differences are located in the N and C termini, the loop immediately preceding B3, the B4-B5 β-hairpin, and the L1 loop (Figure 2B), where the sequences among PGRPs are highly diverse (Figure 2C). PGRP-SA contains two disulfide bridges (Cys11-Cys134 and Cys48-Cys54), whereas PGRP-LB has only one. The highly conserved disulfide bridge Cys48-Cys54 is the target of the *PGRP-SA^seml^* mutation in which Cys54 is changed into a tyrosine (Michel et al. 2001). The Cys48-Cys54 bridge tethers the H2 helix to the central β sheet through the L1 loop (Figure 2A). The other disulfide bond, between Cys11 and Cys134, is solvent exposed and anchors the N-terminal portion of PGRP-SA onto the H3 helix. As the C terminus of the protein is also tethered by insertion of the proline ring of the terminal residue Pro177 into a hydrophobic pocket formed by Ile148 of B8 and Val153 of H4, the structure of PGRP-SA appears to be more compact than that of PGRP-LB. The integral domain structure of PGRP-SA may be required for protein stability, considering that PGRP-SA is an extracellular protein secreted into the *Drosophila* hemolymph. The disulfide bridge Cys11-Cys134 may also be present in several mouse and human PGRPs (Figure 2C).

The most prominent feature of PGRP-SA is a long surface groove demarcated by residues of the H2 helix from one side and of the L1–L4 loops from the other side, with the residues from B3, B4, and B7 of the central β sheet forming the base of the groove (Figure 2A). These residues are among the least conserved, and even the lengths of the L1 and L3 loops vary among members of the PGRP family (Figure 2C). Therefore, the surface groove structure of PGRP-SA is distinct from that of PGRP-LB despite their overall structural similarity (Figure 3A and 3B). The presence of a surface groove on PGRP-SA suggests that it may have a role in PG binding. We performed mutagenesis and functional analysis to test this hypothesis. In the following text, mutations are identified by the one-letter code for the residue in wild-type PGRP-SA, followed by the position of the residue in the amino acid sequence and the one-letter code for the residue to which it was mutated (e.g., S158A has serine in position 158 mutated to alanine). Residues on the surface groove whose side chains are solvent accessible were chosen for mutational analysis (see Figure 2C, 3A, and 3C). These residues are located in three different subregions of the putative docking groove. The first group of residues constitutes the right-side wall of the groove, based on the front view shown in Figure 3 (Tyr64, His65, Asp70, Phe71, and Asn72). The second group is located on the left-side wall of the groove (Val44, Thr45, Tyr100, Ile154, and Ser158). The last group includes Ser75, which sits at the base of the groove. Based on the structure, we also made a Thr-to-Tyr mutation for residue 156; we reasoned that the introduced bulky side chain of Tyr would prevent access of PG to the putative docking groove. In addition, the single mutation I14A, located on the backside of the molecule, was made as a control. None of the residues chosen for mutagenesis is involved in extensive packing interactions. Hence, alterations of these residues are not expected to disrupt the tertiary structure of PGRP-SA. Our hypothesis was that, if the surface groove is indeed involved in PG recognition, the Ala mutations within the groove should exhibit reduced or altered PG-binding activities, whereas the T156Y mutation should completely abolish PG interaction.

**Figure 4:**
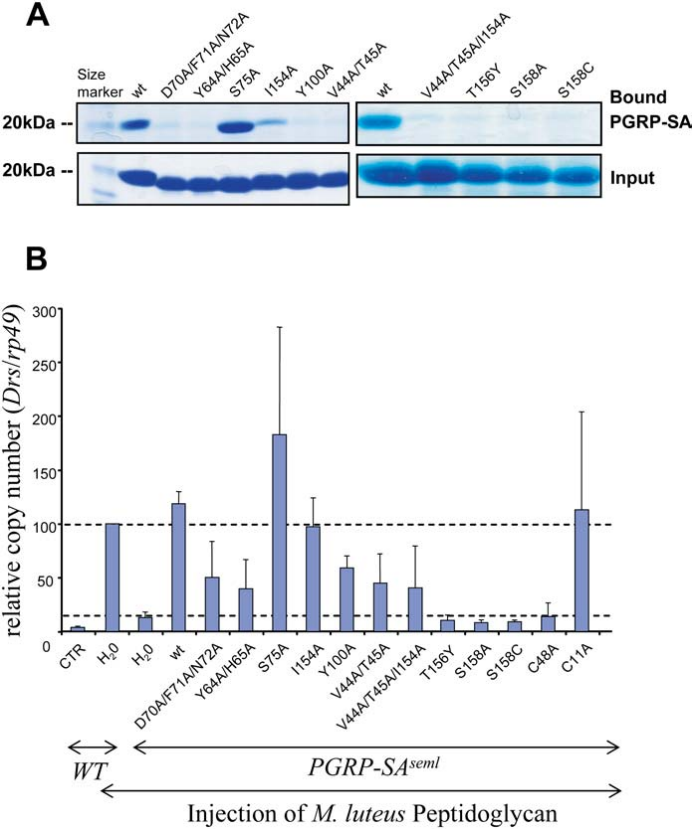
Mutational Analysis of PGRP-SA (A) Upper panel shows the wild-type and mutant rPGRP-SA pulled down by lysine-type PG from *M. luteus.* Lower panel (Input) shows the corresponding protein samples (20 μg) without incubation with PG. (B) The relative Toll signaling activities of the rPGRP-SA mutants. At least three repeats were performed for each experiment. Each bar represents the mean with the standard deviation. The values obtained for the wild type after M. luteus PG injection were arbitrarily set to 100 (upper dashed line). The background activity level is indicated by the lower dashed line. CTR, unchallenged control.

We analyzed the ability of these single- or multiple-Ala mutants to bind lysine-type PG from M. luteus by in vitro PG-binding assays (Figure 4A). The in vivo activity of these rPGRP-SA mutants was examined by analyzing their capacity to rescue the *PGRP-SA^seml^* mutation in the assay described earlier; in addition, the *Drosomycin* expression was measured by quantitative real-time PCR analysis (Figure 4B). These studies showed that mutations at almost every position tested on both walls of the groove region led to impaired PG binding and Toll signaling activity except the S75A mutant, which exhibited an enhanced PG-binding ability. The T156Y mutation, as expected, resulted in a complete loss of the interaction with PG (Figure 4A); as a result, the mutant protein failed to activate the Toll pathway (Figure 4B). Notably, the single mutations S158A and S158C also completely abolished the function of the protein both as a PG recognition receptor and as a Toll activator. The enhanced activity on both PG binding and Toll activation of the S75A mutants suggests that the removal of the hydroxyl group of Ser75 may create a better binding surface for the PG. In fact, Ala and Gly are commonly found at this position in the sequences of PGRPs (see Figure 2C). As expected, the I14A mutation on the backside of the molecule did not affect PG binding or Toll activation (unpublished data). It is interesting that some mutants of PGRP-SA with apparent PG-binding deficiency, for example Y100A and V44A/T45A, could still induce *Drosomycin* expression upon injection in response to challenge with PG from *M. luteus.* This discrepancy may be the result of different sensitivities between the gel-based PG-binding assay, which examines plain physical interaction between PGRP-SA and PG, and the rescue assay, by which the amplified signaling outcome of PG interaction with PGRP-SA, namely *Drosomycin* expression, is observed. Nevertheless, these results together indicate that the surface groove of PGRP-SA mediates both interaction with PG and activation of the Toll pathway. These studies further underscore the role of PGRP-SA as a true pattern recognition receptor, as they demonstrate the correlation between the biochemical recognition of PG and Toll activation through PGRP-SA.

The *PGRP-SA^seml^* mutation results in a Cys-to-Tyr mutation at position 54, which is engaged in a highly conserved Cys48-Cys54 disulfide linkage (Michel et al. 2001). We conducted mutational analysis to investigate whether the *PGRP-SA^seml^* mutation eliminates PGRP-SA function by disrupting the conserved disulfide bridge and thus affecting the protein stability, or by sterically blocking the surface groove with the bulky side chain of Tyr. We found that the C48A mutant failed to be expressed in the insect cell culture, in which all the other wild-type and mutant proteins were expressed (unpublished data). As a result, the culture medium from the C48A mutant failed to restore the *PGRP-SA^seml^* phenotype after injection in *Drosophila* (Figure 4B). By contrast, the C11A mutation, which disrupts the Cys11-Cys134 disulfide bond on the backside of the molecule, had little effect on the function of the protein (Figure 4B). These results suggest that the *PGRP-SA^seml^* mutation disrupts the proper folding of PGRP-SA rather than PG interaction via the docking groove.

It is intriguing that both of the single mutants, S158A and S158C, fail to bind lysine-type PG and to activate Toll. The S158 residue is located on one wall of the docking groove. Seven of the *Drosophila* PGRPs, including PGRP-SC1B and PGRP-LB, have been suggested to possess amidase activity. These “amidase PGRPs” all have a Cys residue at this position, which appears to participate in the Zn coordination in the active site. Substitution of Cys to Ser or Ala in PGRP-SC1B eliminates its enzymatic activity but not its capacity to bind PG, suggesting that the Cys is required for amidase activity (Mellroth et al. 2003). Our data show that, in PGRP-SA, Ser158 is essential for its interaction with PG. Also, Ser158 is highly conserved among PGRPs that have lost the amidase catalytic residues (see Figure 2C). The drastic loss-of-function effect of S158A and S158C mutations suggests that the chemical property of the Ser side chain at this position may be critical for function.

**Figure 5:**
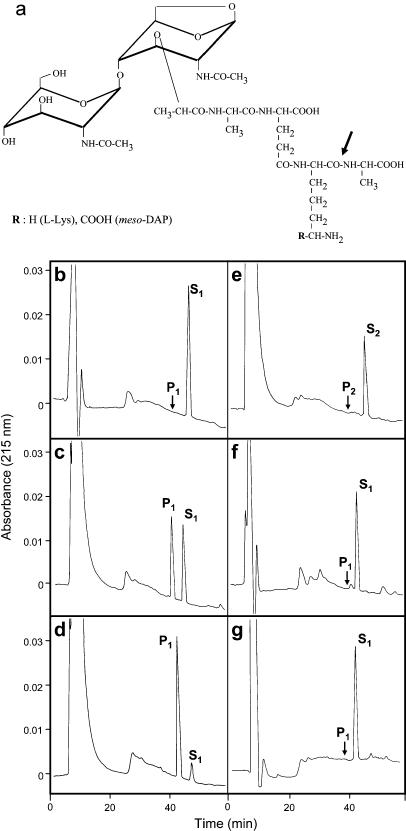
PGRP-SA Is an L,D-Carboxypeptidase (A) Chemical structures of the DAP-type muropeptide, GlcNAc-MurNAc(anhydro)-L-Ala-γ-D-Glu-*meso*-**DAP**-D-Ala, and lysine-type muropeptide, GlcNAc-MurNAc(anhydro)-L-Ala-γ-D-Glu-L-**Lys**-D-Ala, used in the enzymatic assays (substrates S_1_ and S_2_, respectively). The arrow indicates the site of cleavage of the DAP-type substrate S_1_ by the L,D-carboxypeptidase activity. (B–G) Reverse-phase HPLC analysis. Cleavage of the DAP-type substrate S_1_ by wild-type rPGRP-SA results in the generation of GlcNAc-MurNAc(anhydro)-L-Ala-γ-D-Glu-*meso*-DAP (P_1_ product). The position of the peak corresponding to standard GlcNAc-MurNAc(anhydro)-L-Ala-γ-D-Glu-L-Lys (P_2_ product, not generated by rPGRP-SA) is indicated. (B) Incubation of S_1_ without rPGRP-SA for 40 h. (C and D) Incubation of S_1_ with rPGRP-SA for (C) 24 h and (D) 40 h. (E) Incubation of S_2_ with rPGRP-SA for 70 h. (F and G) Incubation of S_1_ with the (F) S158C and (G) H42A mutants for 40 h.

**Figure 6:**
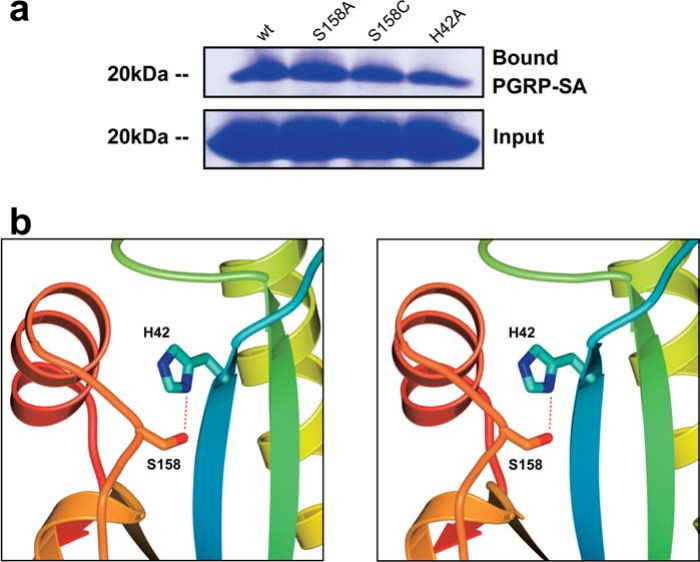
DAP-Type PG-Binding Activities of the S158A/C and H42A Mutants and the Structure of the S158-H42 Dyad in the Active Site (A) Upper panel shows the wild-type and mutant rPGRP-SA pulled down by DAP-type PG from *E. coli.* Lower panel (Input) shows the corresponding protein samples (20 μg) without incubation with PG. (B) Stereo diagram showing the putative active-site residues. Prepared with Bobscript, GL_RENDER, and POV-Ray.

Recently, muropeptides have been identified as the bacterial molecular patterns detected by Nod proteins (Chamaillard et al. 2003; Girardin et al. 2003a, 2003b; Inohara et al. 2003). Free muropeptides are found within bacterial cells as they are constantly synthesized de novo or hydrolyzed from PG and recycled during cell division (Goodell 1985; Goodell and Schwarz 1985); they could be released from bacterial cells during infection and exploited for bacterial sensing by pattern recognition molecules in the host. PGRPs are structural homologues of the *N*-acetylmuramoyl-L-alanine amidase superfamily of proteins, including AmpD and T7 lysozyme, which can hydrolyze monomeric muropeptides or larger PG fragments (Inouye et al. 1973; Kang et al. 1998; Liepinsh et al. 2003; Mellroth et al. 2003). Therefore, muropeptides or their peptidic moieties may serve similarly as specific ligands for PGRP-SA and PGRP-LC via interaction with the PG docking groove. Accordingly, our structural modeling has indicated that the structure of the long docking groove on PGRP-SA is able to fit a ligand with elongated conformation, which a muropeptide or its stem peptide could adopt (unpublished data). Previously, PGRP-SC1B and PGRP-LB have been demonstrated to display a T7 lysozyme-like amidase activity (Kim et al. 2003; Mellroth et al. 2003). We sought to determine if rPGRP-SA has any enzymatic activity, although it has been believed not to possess such an activity; PGRP-SA is missing a critical cysteine residue found in the active site of these amidase PGRPs (Mellroth et al. 2003) (see Figure 2C). We incubated rPGRP-SA separately with either the lysine-type muropeptide, GlcNAc-MurNAc(anhydro)-L-Ala-γ-D-Glu-L-Lys-D-Ala, or the corresponding DAP-type muropeptide, GlcNAc-MurNAc(anhydro)-L-Ala-γ-D-Glu-*meso*-DAP-D-Ala, and analyzed the reaction mixtures afterwards by high performance liquid chromatography (HPLC) (Figure 5). To our surprise, we observed within 40 h of incubation a near-complete cleavage of the DAP-type muropeptide, but not the lysine-type compound, at a specific peptide bond position, resulting in a product consisting of the tripeptide derivative GlcNAc-MurNAc(anhydro)-L-Ala-γ-D-Glu-*meso*-DAP, with the release of the terminal D-Ala (Figure 5B to 5E). These results demonstrated that rPGRP-SA had cleaved between the *meso*-DAP at position 3 and the D-Ala at position 4 on the stem peptide and thus exhibited an L,D-carboxypeptidase activity. Typical Michaelis-Menten kinetics were observed in the substrate concentration range considered (10–500 μM). The *K*_m_ value of rPGRP-SA for its substrate GlcNAc-MurNAc(anhydro)-L-Ala-γ-D-Glu-*meso*-DAP-D-Ala was 21.4 ± 1.8 μM and the catalytic constant *k*_cat_ was 0.48 ± 0.02 h^−1^. The small turnover number of PGRP-SA estimated for hydrolyzing DAP-PG substrates is comparable to the *k*_cat_ of small G proteins such as Ras GTPases. We also tested different other PG-related tetrapeptide compounds as substrates and found that rPGRP-SA hydrolyzed the same peptide bond in DAP-containing muropeptides but had no detectable activity on all the lysine-type compounds tested (unpublished data). This enzymatic activity of rPGRP-SA was not inhibited by ethylenediaminetetraacetic acid (EDTA) or by phenylmethylsulfonyl fluoride (PMSF) (unpublished data). We observed that the two Ser158 mutants, S158C and S158A, did not exhibit any detectable activity (Figure 5F and unpublished data), although both bind DAP-PG from E. coli as wild-type rPGRP-SA does (Figure 6A). In the PGRP-SA structure the Oγ of the Ser158 residue is positioned within hydrogen-bonding distance (2.95 Å) of the Nδ1 of the highly conserved His42 residue (Figure 6B). The fact that the enzymatic activity of rPGRP-SA can be completely eliminated by removing the hydroxyl group of Ser158 (S158A) or by replacing it with a thiol group (S158C) suggests that Ser158 is involved in catalysis rather than in the binding of the DAP-containing substrates. In fact, a Ser-His catalytic dyad of a catalytic antibody was found to be sufficient for catalyzing the hydrolysis of amino acid esters (Zhou et al. 1994). To test this hypothesis, we generated a H42A mutant and analyzed its enzymatic activity. Indeed, this mutant is incapable of hydrolyzing DAP-peptide substrate, although it preserves the ability to bind DAP-PG (see Figure 5G and 6A). Therefore, this result supports the catalytic role of the S158-H42 dyad for the hydrolyzing activity of PGRP-SA. Our enzymatic analysis data show that rPGRP-SA is a carboxypeptidase with an apparent specificity for the L,D-configured DAP-peptide bond between the carboxyl group at the L-center of the *meso*-DAP and the amino group of the following D-Ala residue. Through showing that DAP-containing muropeptides are the substrates for the PGRP-SA enzyme, we provide biochemical evidence suggesting that PGRP-SA may recognize a specific monomeric PG fragment. In support of this finding, it has been demonstrated very recently that specific monomeric DAP-PG fragments can activate the Imd pathway via PGRP-LC in flies and in cell culture (Kaneko et al. 2004).

So far, only one L,D-carboxypeptidase, from *E. coli,* has been identified and characterized (Ursinus and Holtje 1994; Templin et al. 1999). Hence, we report here the first eukaryotic protein exhibiting such an activity specific for peptide bonds existing only in prokaryotes. The DAP-PG hydrolyzing activity of PGRP-SA has a rather slow turnover number. It would be interesting to see if this low intrinsic DAP-PG hydrolyzing activity could be stimulated by another hemolymph protein(s) that could associate with PGRP-SA, such as Gram-negative bacteria-binding protein 1 (GNBP1) (see below). Our observation that a pattern recognition receptor has enzymatic activity is unexpected. However, as rPGRP-SA binds to both lysine-type and DAP-type PGs (see Figure 1F), this finding suggests that the specific activation of Toll by lysine-type PG is achieved by the concomitant ability of PGRP-SA to recognize lysine-type PG and to hydrolyze DAP-type PG. Since the latter has been identified as a strong inducer of the Imd pathway, it will be interesting to see whether the L,D-carboxypeptidase activity of PGRP-SA can influence the Imd pathway positively by generating specific PG fragments that are recognized by PGRP-LC or negatively by scavenging DAP-PG to eliminate its immune-elicitor activity. As PGRP-LCx/a, the other known pathogen-sensing receptors, and several other PGRPs also possess a Ser residue at the position equivalent to S158 in PGRP-SA (see Figure 2C), it will be interesting to see if they possess similar enzymatic activity.

In the present study, we characterized the PG docking groove of PGRP-SA through a combined structural and functional analysis, and we showed that this surface groove mediates both PG sensing and Toll signaling and that Ser158 in the groove is involved in PG interaction, Toll activation, and the newly discovered L,D-carboxypeptidase activity. The S158C mutation has a dramatic negative effect on the ability to activate the Toll pathway. This suggests that the hydroxyl group of Ser158 may mediate critical interaction, perhaps via a hydrogen bond, with a specific lysine-type PG fragment and that this interaction may contribute the bulk of the binding energy. However, we found that the same mutation did not affect binding to DAP-type PG, suggesting that Ser158 is not critical for DAP-type PG interaction (Figure 6A). Therefore, PGRP-SA appears to employ different binding modes for interactions with lysine-type PG versus DAP-type PG. *meso*-DAP differs from L-lysine only by the substitution of a carboxyl group on the Cɛ with D-chirality. As the carboxypeptidase activity of PGRP-SA can act only on DAP-type and not on lysine-type PG compounds, it is likely that the carboxyl group at the D-center of DAP provides the critical interaction(s) with the docking groove residue(s) to help orient the peptide bond between DAP and D-Ala. However, understanding the structural basis of the selectivity to DAP-PG over lysine-PG would require a cocrystal structure of PGRP-SA and a lysine-type PG ligand. As the PG docking groove is lined with residues that are highly diverse among different PGRPs, indicating that each PGRP protein may bind to a specific set of PG fragments, determining the structure of PG ligand-bound PGRP-SA will also provide important insights into PG recognition specificity of this family of proteins. However, so far, no cocrystal of a PGRP protein with a PG compound has been obtained.

*Drosophila* possesses a high number of genes encoding serine proteases and serine protease inhibitors (serpins) (Rubin et al. 2000). Serine protease cascades, operating through sequential zymogen activation, have been implicated in dorsal-ventral fate determination and hemolymph clotting in arthropods (Krem and Cera 2002). A hemolymph serine protease (Persephone) has been shown to mediate the cleavage of Spätzle in response to fungal infection (Ligoxygakis et al. 2002). As PGRP-SA is not involved in fungal-dependent cleavage of Spätzle and activation of Toll, it is believed that this hemolymph pattern recognition protein activates another unidentified proteolytic enzyme(s), resulting in the cleavage of Spätzle specifically in response to bacterial infection. Recently, another hemolymph protein, GNBP1, has been shown to critically participate in activating Toll, perhaps by associating with PGRP-SA, in response to Gram-positive bacterial infection (Gobert et al. 2003; Pili-Floury et al. 2004). Based on our result indicating that PGRP-SA may recognize monomeric PG ligands, it is likely that docking of the specific PG compound onto the surface groove of PGRP-SA may create a new molecular surface that would allow interaction with other Toll-activating factors such as GNBP1. Furthermore, perhaps a multiprotein complex involving PG ligand-bound PGRP-SA and GNBP1 is involved in direct proteolytic activation of the upstream protease of a Spätzle-processing protease cascade. Alternatively, a PG-dependent PGRP-SA/GNBP1 complex may be involved in binding and sequestering a serpin to release the inhibition of the Spätzle-processing enzyme cascade. A hemolymph serpin (Necrotic) has been implicated in inhibiting the proteolytic cleavage of Spätzle upon fungal infection (Levashina et al. 1999). Although a better understanding of PGRP-SA/GNBP1-activated cleavage of Spätzle will require identification of critical players that link the microbial recognition to the proteolytic activation of Spätzle, more detailed biochemical and structural studies on the minimal PG moiety recognized by PGRP-SA and the interaction between PGRP-SA, its specific PG ligand, and GNBP1 are necessary to help define the molecular mechanism of PG recognition mediated by these pattern recognition receptors.

## Materials and Methods

### 

#### Protein expression, purification, and crystallization

Details on the cloning, expression, purification, and crystallization of recombinant *Drosophila* PGRP-SA will be presented elsewhere. Briefly, full-length PGRP-SA (including its N-terminal signal peptide) with a 6×His tag at the C terminus was overexpressed in insect Hi-5 cells using the Bac-to-Bac baculovirus expression system (Invitrogen, Carlsbad, California, United States) and purified with Talon metal affinity resins (Clontech, Palo Alto, California, United States) followed by size exclusion on a Superdex 75 column (Pharmacia, New York, New York, United States) pre-equilibrated in 20 mM Tris-HCl (pH 7.8) and 300 mM NaCl. The purified protein was analyzed by N-terminal sequencing and mass spectrometry to ensure its identity and purity. Crystallization was carried out at 21 °C by the hanging-drop vapor diffusion technique. The protein formed plate-like clusters over a reservoir containing 2.0 M NaKPO_4_ (pH 6.2). Single crystals were produced by two successive rounds of streak- and macroseeding and were cryoprotected in reservoir solution supplemented with 30% glycerol before data collection.

#### Structure determination

We collected X-ray diffraction data using synchrotron radiation at the 19-ID beamline at APS. The diffraction images were processed and scaled with the HKL2000 package (Otwinowski and Monor 1997). The positions of the two molecules in the asymmetric unit were determined by molecular replacement with the program AmoRe (Navaza 1994) using the PGRP-LB structure as the search model (PDB code 1OHT). The two solutions were related by rotation and translation operations, generating a nonsymmetric dimer. The current model was refined after iterative cycles of manual rebuilding with the program O (Jones et al. 1991) and refinement with the program CNS (Brunger et al. 1998) (Table 1). The PGRP-SA dimer in the crystal is probably not biologically relevant, as it was not revealed by gel-filtration chromatography; moreover, the dimer interface was found to involve several phosphate ions from the crystallizing reagent and the first His residue of the affinity tag from one of the monomers.

**Table 1:**
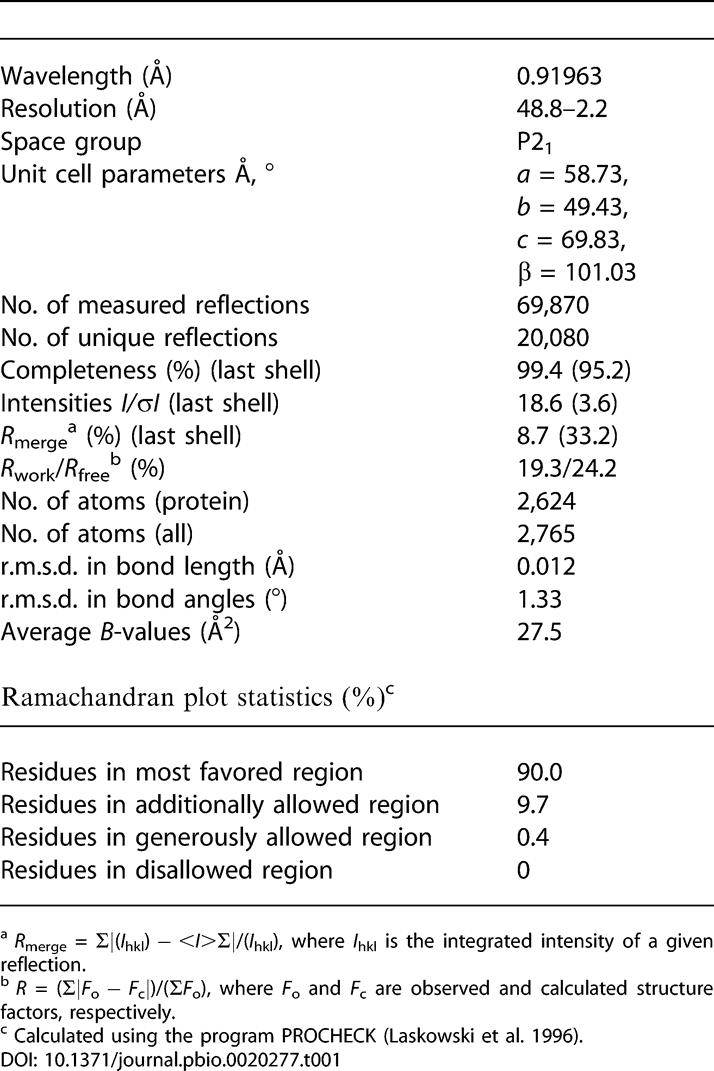
Data Collection and Refinement Statistics

^a^ *R*_merge_ = Σ|(*I*_hkl_) − <*I*>Σ|/(*I*_hkl_), where *I*_hkl_ is the integrated intensity of a given reflection

^b^ *R* = (Σ|*F*_o_ − *F*_c_|)/(Σ*F*_o_), where *F*_o_ and *F*_c_ are observed and calculated structure factors, respectively

^c^ Calculated using the program PROCHECK (Laskowski et al. 1996)

#### Site-directed mutagenesis

Point mutations were generated by a PCR-based strategy using the QuikChange Kit (Stratagene, La Jolla, California, United States), and the identities of the mutagenized products were verified by sequencing.

#### Fly stocks and protein microinjection

y, w, P(*ry^+^, Diptericin-lacZ*), P(*w^+^, Drosomycin-GFP*) flies were used as wild-type strains (Manfruelli et al. 1999). *Drosomycin-GFP, PGRP-SA^seml^* is a line carrying the *semmelweis* mutation in *PGRP-SA* (C54Y) (Michel et al. 2001). *Drosophila* stocks were maintained at 25 °C with standard medium.

A quantity of 9.2 nl of water or rPGRP-SA protein was injected into the thorax of wild-type or *PGRP-SA^seml^* female adults (3–4 d old) using a Nanoject apparatus (Drummond, Broomall, Pennsylvania, United States). One hour later, flies were infected with a thin needle previously dipped into a concentrated culture of M. luteus or given an injection of 9.2 nl of M. luteus PG (5 mg/ml). Flies were then incubated for 24 h at 25 °C. A highly purified solution of M. luteus PG was produced and injected in flies as described by Leulier and colleagues (2003).

#### Quantitative real-time PCR

For *Drosomycin* quantification from whole animals, RNA was extracted using RNA TRIzol (Invitrogen). cDNAs were synthesized using SuperScript II (Invitrogen) and PCR was performed using dsDNA dye SYBR Green I (Roche Diagnostics, Basel, Switzerland) on a Lightcycler (Roche). All samples were analyzed in duplicate and the amount of mRNA detected was normalized to control Rp49 mRNA values. We used normalized data to quantify the relative levels of a given mRNA according to cycling threshold analysis (ΔCt).

#### PG-binding assay

The assay was performed at 4 °C by incubating 20 μg of purified wild-type or mutant rPGRP-SA with 300 μg of insoluble PGs, prepared as described previously (Leulier et al. 2003), in 300 μl of binding buffer containing 20 mM Tris-HCl (pH 7.8) and 300 mM NaCl on a shaking platform for 1 h. Bound protein, retained in the PG pellet after spinning the incubation mixture at 16,000 × *g* for 5 min, was washed with 1 ml of binding buffer followed by a 5-min spin and finally dissolved in 10 μl of SDS buffer for sodium dodecyl sulfate polyacrylamide gel electrophoresis (SDS-PAGE) analysis. The PG-bound rPGRP-SA was visualized by Coomassie Blue staining.

#### Enzymatic assay and reverse-phase HPLC analysis

The activity was tested in 20 mM HEPES (pH 7.4) containing 2.5 mM EDTA, 50 μM substrate, and enzyme (25 μg of wild-type or mutant rPGRP-SA) in a total volume of 50 μl. After incubation for the indicated period at 37 °C, the mixture was injected on a Nucleosil 100 C_18_ 5μ reverse-phase HPLC column (4.6 mm × 250 mm, Alltech France, Templemars, France) and elution was performed at 0.6 ml/min with buffer A (50 mM sodium phosphate [pH 4.45]) for 10 min and then with a gradient of methanol in buffer A (from 0% to 25% in 50 min). Peaks were detected at 215 nm. In all cases, substrates and products were purified and desalted by HPLC, and their identity was confirmed by amino acid and mass spectrometry analyses.

## Supporting Information

The atomic coordinates and structure factors have been deposited in the Protein Data Bank (http://www.rcsb.org/pdb/) under accession number 1S2J.

## Abbreviations

APS: Advanced Photon Source
DAP: diaminopimelic acid
EDTA: ethylenediaminetetraacetic acid
GFP: green fluorescent protein
GlcNAc: *N*-acetyl glucosamine
GNBP1: Gram-negative bacteria-binding protein 1
HPLC: high performance liquid chromatography
Imd: immune deficiency
MurNAc: *N*-acetyl muramic acid
PG: peptidoglycan
PGRP: peptidoglycan recognition protein
*PGRP-SA^seml^*: PGRP-SA deficient
PMSF: phenylmethylsulfonyl fluoride
r.m.s.d.: root-mean-square deviation
rPGRP-SA: recombinant PGRP-SA
SDS-PAGE: sodium dodecyl sulfate polyacrylamide gel electrophoresis
serpin: serine protease inhibitor
*wt*: wild type
